# Comparison of the incidence of fetal prolonged deceleration after induction of labor analgesia between dural puncture epidural and combined spinal epidural technique: a pilot study

**DOI:** 10.1186/s12884-023-05473-0

**Published:** 2023-03-16

**Authors:** Shoko Okahara, Rie Inoue, Yumi Katakura, Hitomi Nagao, Saori Yamamoto, Shuko Nojiri, Jun Takeda, Atsuo Itakura, Hiroyuki Sumikura

**Affiliations:** 1 Department of Anesthesiology, Juntendo University Hospital, 3-1-3 Hongo Bunkyo-ku Tokyo, 113-8431 Tokyo, Japan; 2 Clinical Research Support Center, Juntendo University, 2-1-1 Hongo Bunkyo-ku Tokyo, 113-8421 Tokyo, Japan; 3 Department of Obstetrics and Gynecology, Juntendo University Hospital, 3-1-3 Hongo Bunkyo-ku Tokyo, 113-8431 Tokyo, Japan

**Keywords:** Abnormal cardiotocogram tracing, Combined spinal epidural analgesia, Dural puncture epidural, Labor analgesia, Non-reassuring fetal status

## Abstract

**Background:**

Abnormal cardiotocogram (CTG) tracing may appear after induction of neuraxial labor analgesia. Non-reassuring fetal status (NRFS) indicated by severely abnormal tracings, such as prolonged deceleration (PD) or bradycardia, can necessitate immediate operative delivery. Combined spinal epidural analgesia (CSEA) is known to result in more frequent abnormal tracings than epidural analgesia (EA); however, the corresponding data related to dural puncture epidural (DPE) are unclear. We aimed to evaluate the rates of incidence of severe abnormal CTG after induction of DPE and CSEA.

**Methods:**

In this study of nulliparous women with full-term pregnancy, data for the DPE intervention group were prospectively collected, while those for the CSEA control group were obtained from medical records. Neuraxial analgesia was performed with cervical dilation ≤ 5 cm, administering initial epidural dosing of 15 mL of 0.125% levobupivacaine with fentanyl 2.5µg/mL for DPE, and intrathecal 0.5% bupivacaine 2.5 mg (0.5ml), fentanyl 10 µg (0.2ml), and 1.3 mL of saline for CSEA. The primary outcome was the incidence of PD, defined as a fetal heart rate reduction ≥ 15 bpm below the baseline and with a lowest value < 80 bpm, and lasting for ≥ 2 min but < 10 min (fetal heart rate < 80 bpm does not have to last for ≥ 2 min), within 90 min after induction of neuraxial labor analgesia.

**Results:**

A total of 302 patients were analyzed, with 151 in each group. The incidence of PD after DPE induction was significantly lower than that after CSEA induction (4.0% vs. 14.6%, *P* = 0.0015, odds ratio = 0.243, 95% confidence interval = 0.095–0.617).

**Conclusion:**

DPE appears to be a safer method compared to CSEA for neuraxial labor analgesia in the early stages of labor for nulliparous women.

**Trial registration:**

UMIN-CTR: UMIN000035153. Date registered: 01/01/2019.

## Introduction

Abnormal cardiotocogram (CTG) tracing, which suggests a non-reassuring fetal status (NRFS) after induction of neuraxial labor analgesia, is a major concern in obstetric anesthesia. Despite the existing consensus that neuraxial labor analgesia does not increase the cesarean delivery rate [1, 2], preparation for immediate operative delivery and intrauterine fetal resuscitation is essential in such cases. Therefore, the association between neuraxial labor analgesia and abnormal CTG tracing has been widely investigated. Abnormal CTG is observed more frequently after induction of combined spinal epidural analgesia (CSEA) when compared to epidural analgesia (EA) alone [3, 4].

The incidence of abnormal CTG tracing in previous studies ranged from 4.9 to 31.7% [5–7]. One of the reasons for this broad variation may be the differences in the definition of abnormal CTG tracing across studies. In general, the broader the definition of abnormal CTG tracing, the lower the proportion of cases requiring urgent obstetric intervention, whereas a narrower definition will be associated with a higher proportion of cases requiring urgent obstetric intervention. Prolonged deceleration (PD) and bradycardia are more likely to necessitate immediate operative delivery. Abrao et al. [5] compared the incidence of PD and bradycardia as primary outcomes between CSEA and EA and found that they were observed significantly more frequently with CSEA.

Dural puncture epidural (DPE) has recently been highlighted as a novel technique in the field of obstetric anesthesia. DPE is a modified form of CSEA that uses a needle-through-needle technique to puncture the dura with a spinal needle without administering the drug into the subarachnoid space. Instead, a small amount of the solution administered through the epidural catheter is expected to transfer to the subarachnoid space via a dural hole. DPE performed as a labor analgesia technique offers higher analgesic quality than EA, including faster onset, better sacral analgesia, and less unilateral blockage [8, 9], and causes fewer adverse reactions, such as maternal pruritus and hypotension, than CSEA [9]. However, data for abnormal CTG tracing related to DPE are limited. A systematic review that compared DPE and EA included too few studies to draw a conclusion [10]. To the best of our knowledge, only one study has compared DPE with CSEA [7], and the authors reported that CTG tracing as a secondary outcome was significantly less likely to deteriorate from category I to II (in the 3-tier NICHD system) in DPE than in CSEA.

We aim to evaluate the hypothesis that PD occurs less frequently after induction of DPE than CSEA by using a specific definition (a fetal heart rate reduction ≥ 15 bpm below the baseline and with a lowest value < 80 bpm, and lasting for ≥ 2 min but < 10 min (fetal heart rate < 80 bpm does not have to last for ≥ 2 min)) for the primary outcome. In addition, various maternal, fetal, and anesthetic-related data were analyzed as secondary outcomes.

## Methods

### Study design

This study was approved by the ethics committee of Juntendo University Hospital, Tokyo, Japan (H18-0187) and registered with UMIN-CTR (UMIN000035153) [11] before initiation. A power analysis was originally performed; however, COVID-19 pandemic that occurred during the study period made it impossible to continue recruiting patients. As a result, the study design was altered to a pilot study with a smaller sample size after consulting a statistical expert (S.N.). The ethics committee’s approval was obtained for the amendment. Data for the DPE intervention group was collected prospectively for the study, whereas data for the CSEA control group was collected from historical cases. For the DPE group, parturients who delivered at Juntendo University Hospital between March 2019 and April 2020 were considered. Nulliparous parturients aged 20–50 years who were eligible for vaginal delivery, showed no contraindication for neuraxial anesthesia, and provided verbal and written consent for the study were eligible for inclusion if they also met the following criteria on admission for delivery: full-term pregnancy (37 to 41 weeks) and cervix dilation ≤ 5 cm.

For the CSEA group, the ethics committee approved exemption from informed consent, but mandated an opt-out method. That is, the information about the study was published on the hospital’s website, and any applicable parturient who chose not to participate in the study were excluded by informing the facility of their choice. Parturients who had delivered at the institution prior to the study and met the same inclusion criteria as the DPE group were included in the CSEA group. Cases were collected from April 1, 2018, onward, in chronological order, until the number of cases was the same as that for the DPE group.

When CTG was interpreted, cases already showing abnormal CTG tracing within 30 min before induction of labor analgesia were excluded, as were cases involving spontaneous vaginal delivery within 90 min after induction of labor analgesia to provide a minimum length of CTG recordings. Notably, however, cases of emergency cesarean section or instrumental delivery due to PD within 90 min remained eligible for the study.

The medical care provided to parturients in this study was performed in accordance with the “Guideline for the Obstetrical Practice in Japan 2020” by the Japan Society of Obstetrics and Gynecology (JSOG).

### Outcomes

The primary outcome was the incidence of PD within 90 min after induction of neuraxial labor analgesia. We based our definition of PD on that of severe prolonged deceleration according to the five-level fetal heart rate classification system by JSOG [11]: a fetal heart rate reduction ≥ 15 bpm below the baseline and with a lowest value < 80 bpm, and lasting for ≥ 2 min but < 10 min (fetal heart rate < 80 bpm does not have to last for ≥ 2 min). In addition, bradycardia that lasted longer than 10 min was also included as the same primary outcome.

Secondary outcomes included the incidence of deterioration in CTG levels from before to after induction of labor analgesia, mode of delivery, interval between induction and full dilation of the cervix, duration of the first and second stages of labor, 1- and 5-min Apgar scores, umbilical artery blood pH, consumption of local anesthetic during labor, use of physician top-up, and patients’ satisfaction with labor analgesia.

### Protocols for the DPE and CSEA techniques

A protocol for labor analgesia with DPE was established for this study. A needle-through-needle technique was performed to puncture the dura by using a 27-gauge, 130-mm pencil-point spinal needle, Pencan® (B. Braun Aesculap Japan, Tokyo, Japan), inserted into an 18-gauge, 80-mm epidural needle, Perican® epidural cannula (B. Braun Aesculap Japan, Tokyo, Japan). The puncture was confirmed by the flow of the cerebrospinal fluid (CSF). A 20-gauge, 1000-mm multi-orifice catheter, Perifix® Soft-Tip catheter (B. Braun Aesculap Japan, Tokyo, Japan), was then placed into the epidural space. After confirming absence of aspiration of blood or CSF, 5 mL of 0.125% levobupivacaine with fentanyl 2.5 µg/mL was administered via the catheter. Five minutes later, 10 mL of the same solution was administered. The sensory block was confirmed by the cold-loss method with an alcohol swab 15 and 20 min after the initial administration. A 5ml bolus of PCEA content is added by an obstetric anesthesiologist after 40 min from the initial administration, followed by initiation of patient-controlled epidural analgesia (PCEA).

The protocol for the CSEA group was based on institutional practices. An 18-gauge, 80-mm epidural needle and a 27-gauge, 122 mm spinal needle were used for the needle-through-needle technique. Initial spinal dosing consisted of 0.5% bupivacaine 2.5 mg (0.5ml), fentanyl 10 µg (0.2ml), and 1.3 mL of saline. Then, a 20-gauge, 1000-mm multi-orifice catheter was placed into the epidural space. After confirming the absence of aspiration of blood or CSF, a 5ml bolus of PCEA content is added by an obstetric anesthesiologist approximately 30 and 60 min, respectively, after the initial spinal administration, followed by initiation of patient-controlled epidural analgesia (PCEA). All the products used for the CSEA group were obtained from Smith Medical (Minnesota, USA).

The settings for PCEA were equivalent in both groups: 200 mL of 0.08% levobupivacaine with fentanyl 2 µg/ml, no background infusion, a PCA dose of 5 mL, and a lock-out interval of 15 min. Once initial pain relief was achieved, obstetric anesthesiologists visited delivery units approximately every hour to check maternal vital signs, consumption of PCEA, numeric rating scale (NRS) scores, and sensory block, as needed. Intervention for breakthrough pain was left to the discretion of the obstetric anesthesiologists in charge. To administer extra solution from epidural catheter, either manual top-up of local anesthetic with or without fentanyl or a bolus of PCEA content by a physician was used.

### Analysis of CTG

After data collection, CTG data stored in the hospital patient electronic system were retrospectively interpreted by three maternal and fetal specialists certified by the Japan Society of Perinatal and Neonatal Medicine (including one of the co-authors, J.T.). In cases where PD, the primary outcome, occurred, the interval from induction of analgesia to PD and the duration of PD were recorded. The evaluation of CTG was based on the five-level fetal heart rate classification system by JSOG. This classification stratifies CTG tracings into five levels—I:normal, II:benign, III:mild, IV:moderate, and V:severe—depending on the type of variability, baseline, and deceleration of the CTG.

### Statistical analysis

Demographic characteristics and outcomes with continuous variables were compared using Student’s t-test (Satterthwaite) or Mann–Whitney U test depending on the distribution of the data. The chi-square test was used for categorical variables. Statistical significance was set at p < 0.05. Univariate analysis of the primary outcome was performed using logistic regression analysis to obtain odds ratios and 95% confidence intervals. The Kaplan–Meier curve was used to visualize the primary outcome. All statistical analyses were performed using the statistical software SAS Enterprise Guide 8.1.

## Results

**Fig. 1:**
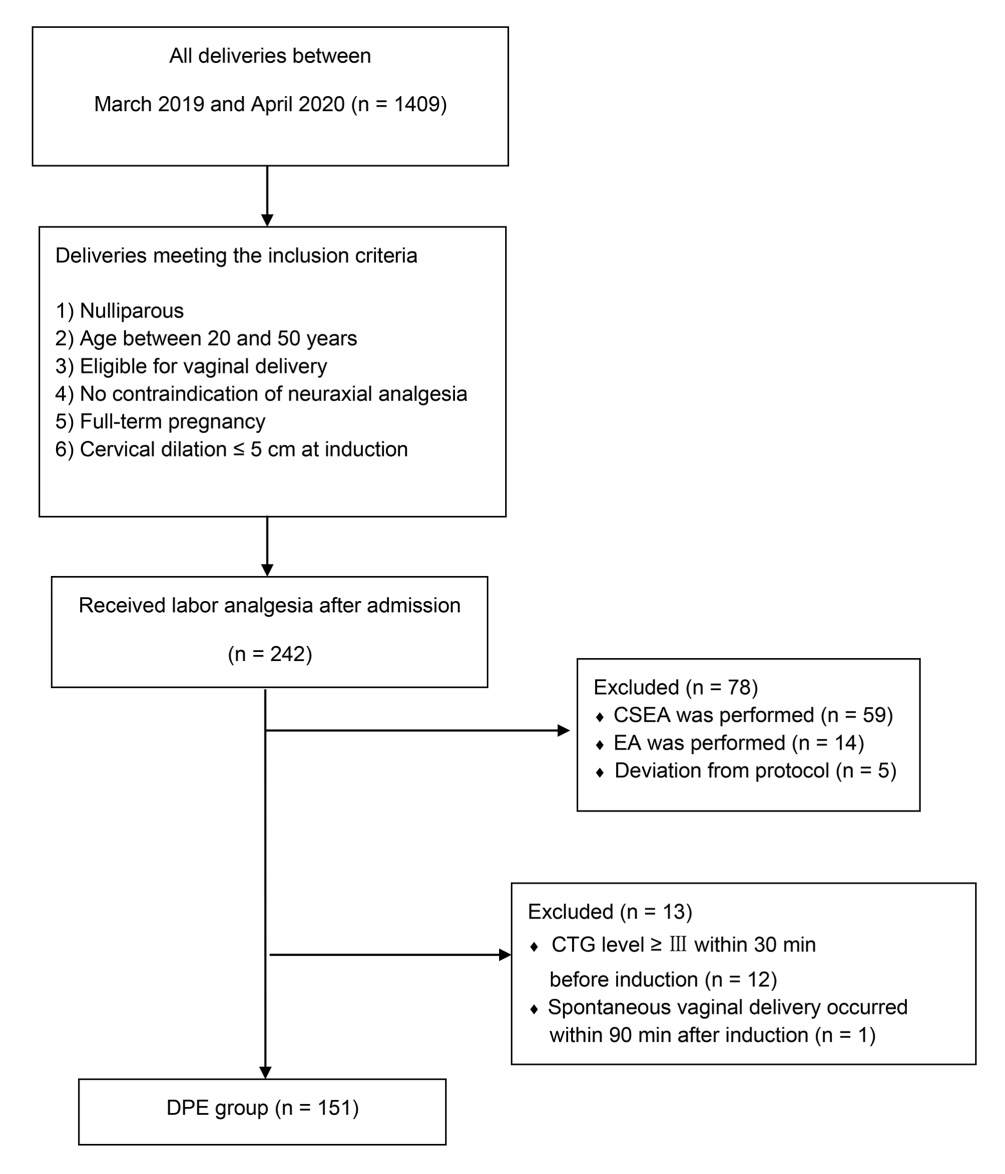
Participant flow in the DPE group Legend: CSEA, combined spinal epidural analgesia; EA, epidural analgesia; CTG, cardiotocogram; DPE, dural puncture epidural

**Fig. 2:**
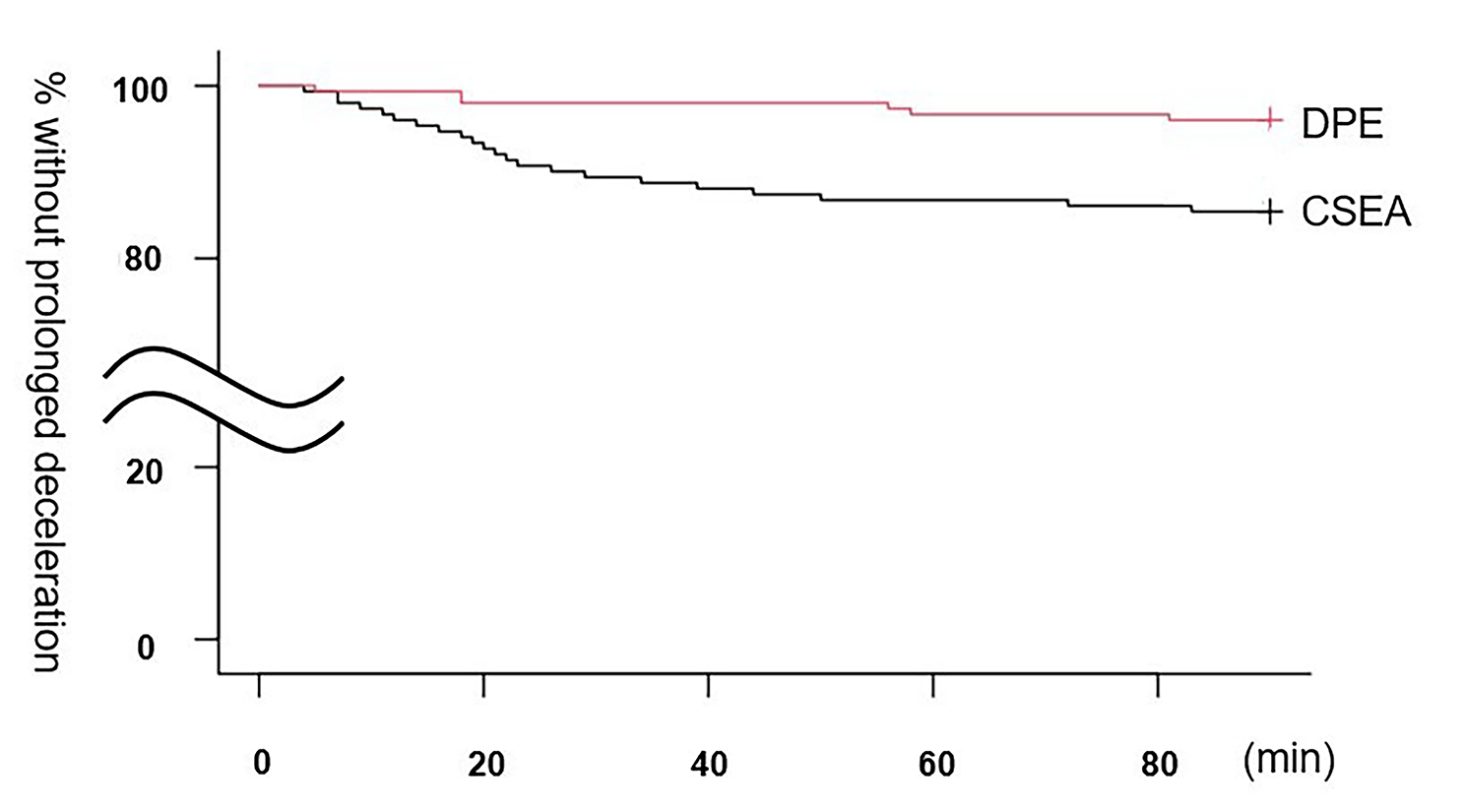
Kaplan-Meier curve illustrating time after induction at which PD occurred Legend: CSEA, combined spinal epidural analgesia; DPE, dural puncture epidural

The participant flow in the DPE group is shown in Fig. 1. Demographic characteristics were similar between the two groups (Table 1). The primary outcome quantifier–the incidence of PD within 90 min after induction of labor analgesia–was significantly lower with DPE than with CSEA (Table 2). Incidence was defined as the ratio of the number of cases showing the primary outcome to the total number of cases. The Kaplan–Meier curve provided a visual representation of the time at which PD occurred (Fig. 2).

The incidence of deterioration in CTG levels from before to after induction of labor analgesia is shown in Table 3. The definition of “deterioration” is when the CTG level (I-V) of the five-level fetal heart rate classification system by JCOG increase after induction. CSEA was associated with more cases in which the CTG showed deterioration after induction, although the level before induction was similarly distributed between the two groups. Mode of delivery; interval from induction to full dilation of the cervix, first stage of labor, and second stage of labor; and the use of uterotonics at induction showed no difference between the two groups (Table 4). Fetal outcomes, including Apgar score (1 and 5 min) and umbilical artery blood pH, were also not significantly different between the groups (Table 4). Parturients in both groups required approximately the same amount of local anesthetic with PCEA during labor; however, the use of physician top-up and patients’ satisfaction with labor analgesia were significantly different between the groups.

**Table 1:** Demographic characteristics

	DPE(*n* = 151)	CSEA(*n* = 151)	*P*-value
Age (yr)	34.0 ± 3.9	34.0 ± 4.6	0.9574
Height (cm)	159.5 ± 5.9	159.2 ± 5.2	0.6884
Weight (kg)	63.3 ± 8.2	63.3 ± 8.7	0.9892
Gestational age (wk)	40.0 ± 1.0	40.1 ± 0.9	0.3863
NRS at induction	8 [7–9]	8 [7–9]	0.2396
Status at induction			
Cervical dilation (cm)	4 [3–4]	3 [3–4]	0.7631
Cervical effacement (%)	60[50–70]	60[50–70]	0.1753
Fetal station	-2[(-3)-(-2)]	-2[(-3)-(-2)]	0.0599
Use of uterotonics at induction (%)	28(18.5)	23(15.2)	0.4425

Values are mean ± standard deviation, median [interquartile range], or *n* (%). *P*-values were obtained by Student’s t-test (Satterthwaite), Mann–Whitney U test, or chi-square test. *P* < 0.05 was considered significant for all outcome comparisons. DPE, dural puncture epidural; CSEA, combined spinal epidural analgesia; NRS, numeric rating scale

**Table 2:** Primary outcome: The incidence of PD within 90 min after induction of labor analgesia

	DPE(*n* = 151)	CSEA(*n* = 151)	*P*-value	Odds ratio	95% CI
PD(+)	6(4.0)	22(14.6)	0.0015	0.243	0.095–0.617
PD(-)	145(96.0)	129(85.4)			

Values are *n* (%). *P*-values were obtained by the chi-square test. Odds ratios and 95% confidence intervals were obtained by univariate logistic regression. DPE, dural puncture epidural; CSEA, combined spinal epidural analgesia; PD, prolonged deceleration; CI, confidence interval

**Table 3:** The incidence of deterioration in CTG levels from before to after induction of labor analgesia

	DPE(*n* = 151)	CSEA(*n* = 151)	*P*-value	Odds ratio	95% CI
Deterioration(+)	67(44.4)	85(56.3)	0.008	0.57	0.359–0.904
Deterioration(-)	83(55.0)	60(39.7)			

Values are *n* (%). *P*-values were obtained by the chi-squared test. DPE, dural puncture epidural; CSEA, combined spinal epidural analgesia

**Table 4:** Secondary outcomes

Outcomes	DPE(*n* = 151)	CSEA(*n* = 151)	*P*-value
Mode of delivery			
ND	80(53)	66(43.7)	0.1377
FD	53(35.1)	70(46.4)	
Em C/S	18(11.9)	15(9.9)	
Duration of delivery			
Induction to full dilation of cervix (min)	378[218–570]	342[215.5–559]	0.6653
First stage of labor (min)	705[470–990]	696[465.5-907.5]	0.4281
Second stage of labor (min)	114.5[60–184]	125[60-173.5]	0.9106
Fetal status			
1-min Apgar score	9 [8–9]	9 [8–9]	0.4729
5-min Apgar score	10 [9–10]	10 [9–10]	0.286
Umbilical cord blood pH	7.3 ± 0.1	7.3 ± 0.1	0.4342
Anesthetic dose			
With PCEA pump	93.2 ± 65.3	99.7 ± 70.8	0.4095
Using physician top-up	51(33.8)	90(59.6)	< 0.0001
Patients’ satisfaction score			
5	63	106	< 0.0001
4	63	32	
3	5	9	
2	0	1	
1	0	0	
Unknown	20	3	-

Values are *n* (%), median[interquartile range], mean ± standard deviation, or n. *P*-values were obtained by the Chi-squared test, Mann–Whitney U test, or Student’s t-test (Satterthwaite). DPE, dural puncture epidural; CSEA, combined spinal epidural analgesia; ND, normal delivery; FD, forceps delivery; Em C/S, emergency cesarean delivery; PCEA, patient-controlled epidural analgesia. Patients’ satisfaction score is 1 for the least satisfied and 5 for the most satisfied

## Discussion

In the present study, DPE was associated with a significantly lower incidence of PD after induction of analgesia than CSEA (4.0% vs. 14.6%, *P* = 0.0015, odds ratio = 0.243, 95% confidence interval = 0.095 to 0.617).

Among the various types of abnormal CTG findings suggestive of NRFS, PD is a particularly concerning finding, since persistent PD can progress to bradycardia and necessitate immediate operative delivery. The cervix needs to be fully open, and the baby’s head should be sufficiently descendant, failing which an emergency cesarean delivery is inevitable. In both situations, obstetric anesthesiologists must respond quickly. Therefore, these severely abnormal CTG tracings should be considered distinct from other decelerations in which careful follow-up is allowed.

Although several studies have compared abnormal CTG tracings between CSEA and EA, few studies have analyzed abnormal CTG tracing limited to PD as the primary outcome between CSEA and EA [5, 6], and their results were not consistent. Skupski et al. [6] defined PD as a fetal heart rate 40 bpm below the baseline or below 90 bpm for 2 min or more, and found no significant difference in the frequency of PD between CSEA and EA. In contrast, Abrao et al. [5] reported that PD and bradycardia, defined as a reduction of 15 bpm or more in the baseline of the FHR lasting > 2 min and < 10 min, and a reduction in the baseline to < 100 bpm, occurred more frequently with CSEA. This discrepancy may be attributable to differences in the definitions of abnormal CTG tracing between the studies. Moreover, the duration of CTG observation may also affect the findings for abnormal CTG tracing. Abrao et al. [5] interpreted CTG within 15 min after induction, whereas Skupski et al. [6] performed evaluations within 90 min. If the duration of CTG observation after induction was too short, the interpretation of CTG may have been completed before initial pain relief with EA was achieved; thus, abnormal CTG tracing, which could have occurred subsequently, would have been missed.

The duration of CTG observation before induction in our study (30 min) was determined in accordance with current institutional practices; if there was obvious PD or bradycardia immediately before induction, we postponed induction for approximately 30 min to confirm reassuring fetal status with CTG tracing. Furthermore, the duration of CTG observation after induction (90 min) was based on the study by Skupski et al. [6]. Although a longer observation period may include abnormal CTG tracing unrelated to anesthesia induction, only four cases showed PD beyond 60 min in our study, and the superiority of DPE remained unchanged even without these cases. Therefore, the post-induction observation time of 60 min may be more preferable in future studies.

As for DPE, no study has explored PD as a primary outcome. In a relatively recent study [12] comparing the frequency of bradycardia (< 110 bpm) as a secondary outcome between DPE and EA, statistical analysis was not performed because of the small sample size (*n* = 40/group). Only one study compared CTG tracing data [7] as a secondary outcome between DPE and CSEA and reported that DPE was associated with a significantly lower rate of deterioration of CTG tracing from category I to II (3-tier NICHD system) after induction of anesthesia. Nevertheless, the relevance of this result to the frequency of immediate obstetric interventions is questionable.

In this study, an evaluation of fetal status using the changes in the CTG tracing data before and after induction were also investigated as secondary outcomes. Although the NICHD system is commonly used for the classification of CTG, its evaluation is based on three levels (normal, intermediate, and abnormal). In contrast, the JSOG five-level fetal heart rate classification system, which is adapted nationwide in Japan, has five tiers (I: normal, II: benign variant, III: mild variant, IV: moderate variant, and V: severe variant), allowing for more sensitive analysis. For instance, PD, the primary outcome in this study, is rated level II in the NICHD three-tier system but is subdivided into levels IV and V according to variability status in the JSOG five-level system. We showed that deterioration occurred more frequently in CTG tracing after induction of anesthesia in CSEA than in DPE (Table 3).

In the present study, six patients showed PD after DPE compared to 22 after CSEA. Of these 28 patients, only three who received CSEA underwent operative delivery directly indicated by PD. Two cases of forceps delivery involved PD 14 and 39 min after induction; both showed a cervical dilation of 4 cm at induction, but forceps delivery could be performed, since the cervix had progressed to full dilation at the time of PD. It was difficult to determine whether PD was associated with CSEA induction or rapid progression of labor. One case of emergency cesarean section showed PD 22 min after induction: cervical dilation remained 8.5 cm at the timing of PD, and category 2 (NICE classification) cesarean section was performed. The DPE group did not include any operative delivery directly indicated by PD within 90 min after induction.

The cesarean section rates in the DPE and CSEA groups were 11.9% and 9.9%, respectively, with no statistically significant difference (Table 4). However, the rate was relatively low, despite the fact that anesthesia was initiated in the early stage of labor with nulliparity. The rate of labor analgesia at our institution was nearly 90%, and immediate cesarean section could be performed anytime. However, in Japan, delivery facilities are not centralized, and approximately half of all deliveries are performed in private clinics where immediate cesarean section is not always possible. Moreover, the rate of labor analgesia nationwide is less than 10%. Thus, when PD occurs after induction in small facilities with little experience of labor analgesia, essential operative delivery may not be performed immediately. Therefore, avoiding abnormal CTG findings indicating NRFS post-induction may be more important in Japan than in other countries where labor analgesia is performed as a normal practice.

DPE required significantly fewer physician top-ups for breakthrough pain than did CSEA (Table 4). This result is consistent with the study by Chau et al., [7] who proposed two possible reasons for this difference. One is that a failed transition from spinal anesthesia to epidural anesthesia in the CSEA group causes breakthrough pain and the request for top-ups. The other reason proposed by Chau et al. is that the induction of CSEA in the early stage of labor is more likely to yield pain than EA because CSEA causes greater uterine contractions and more rapid cervical dilation. Our data showed that the time from induction of anesthesia to full dilation of the cervix was not significantly different between the groups (Table 4); however, uterine hypertonus without abnormal CTG tracing may have induced breakthrough pain.

This study had a few limitations. Firstly, without the prior sample size calculation, the incidence of the primary outcome in the DPE group was too low to apply multivariable logistic regression. Further prospective studies on the basis of the present results are needed to achieve better evidence level. Secondly, maternal and fetal specialists were not blinded to groups during the evaluation of CTGs. In the process of reading the CTGs stored in the hospital patient electronic system, they also had access to information about the deliveries. Thirdly, due to the distinct enrollment periods for the two groups, medical staff was partially different between the two groups. Although the guidelines for medical practice by JSOG have not changed between the two periods, differences in obstetric outcomes could have been influence by the different obstetric intervention. In addition, the use of a 27-gauge spinal needle in this study may be controversial. However, the only available size of spinal needle designed for CSEA with needle-through-needle technique was 27-gauge in Japan at study period. Although the efficacy of DPE with a 27-gauge dural puncture over EA has been questioned in one study [13], a more recent study by Yadav et al. [14] confirmed its advantage. Contreras et al. [15] also reported that DPE with either 25- or 27-gauge spinal needles was qualitatively similar for pain control, with the exception of time to onset. We therefore assume justified in our use of a 27-gauge spinal needle.

## Conclusion

We presented data on the incidence of severe abnormal CTG tracing and PD after induction of DPE in a relatively large sample size of more than 300 cases. PD occurring during labor demands immediate gathering of medical staff, equipment and can necessitate operative delivery in some cases. Induction of neuraxial labor analgesia is one of the potential causes. Our results show that the incidence of PD is reduced under DPE compared to CSEA. We therefore conclude that DPE can be a safer method of neuraxial analgesia than CSEA in nulliparous women in the early stage of labor.

## Data Availability

The datasets generated and/or analyzed during the current study are available from the corresponding author on reasonable request.

## Abbreviations

CI: Confidence interval
CSEA: Combined spinal epidural analgesia
CSF: Cerebrospinal fluid
CTG: Cardiotocogram
DPE: Dural puncture epidural
EA: Epidural analgesia
JSOG: Japan Society of Obstetrics and Gynecology
NRFS: Non-reassuring fetal status
NRS: Numeric rating scale
PCA: Patient-controlled analgesia
PCEA: Patient-controlled epidural analgesia
PD: Prolonged deceleration
